# Review and External Evaluation of Population Pharmacokinetic Models for Vedolizumab in Patients with Inflammatory Bowel Disease: Assessing Predictive Performance and Clinical Applicability

**DOI:** 10.3390/biomedicines13010043

**Published:** 2024-12-27

**Authors:** Marija Jovanović, Ana Homšek, Srđan Marković, Đorđe Kralj, Petar Svorcan, Tamara Knežević Ivanovski, Olga Odanović, Katarina Vučićević

**Affiliations:** 1 Department of Pharmacokinetics and Clinical Pharmacy, Faculty of Pharmacy, University of Belgrade, 11221 Belgrade, Serbia; 2 Department of Gastroenterology and Hepatology, University Hospital Medical Center “Zvezdara”, 11000 Belgrade, Serbia; 3 Faculty of Medicine, University of Belgrade, 11000 Belgrade, Serbia

**Keywords:** predictive performance, simulations, prediction error, NONMEM, vedolizumab, pharmacokinetics

## Abstract

Background/Objectives: Several population pharmacokinetic models of vedolizumab (VDZ) are available for inflammatory bowel disease (IBD) patients. However, their predictive performance in real-world clinical settings remains unknown. This study aims to externally evaluate the published VDZ pharmacokinetic models, focusing on their predictive performance and simulation-based clinical applicability. Methods: A literature search was conducted through PubMed to identify VDZ population pharmacokinetic models. A total of 114 VDZ concentrations from 106 IBD patients treated at the University Medical Center “Zvezdara”, Republic of Serbia, served as the external evaluation cohort. The predictive performance of the models was assessed using prediction- and simulation-based diagnostics. Furthermore, the models were utilized for Monte Carlo simulations to generate concentration–time profiles based on 24 covariate combinations specified within the models. Results: Four published pharmacokinetic models of VDZ were included in the evaluation. Using the external dataset, the median prediction error (MDPE) ranged from 13.82% to 25.57%, while the median absolute prediction error (MAPE) varied between 41.64% and 47.56%. None of the models fully met the combined criteria in the prediction-based diagnostics. However, in simulation-based diagnostics, pvcVPC showed satisfactory results, despite wide prediction intervals. Analysis of NPDE revealed that only the models by Rosario et al. and Okamoto et al. fulfilled the evaluation criteria. Simulation analysis further demonstrated that the median VDZ concentration remains above 12 μg/mL at week 22 during maintenance treatment for approximately 45–60% of patients with the best-case covariate combinations and an 8-week dosing frequency. Conclusions: None of the published models satisfied the combined criteria (MDPE, MAPE, percentages of prediction error within ±20% and ±30%), rendering them unsuitable for a priori predictions. However, two models demonstrated better suitability for simulation-based applications.

## 1. Introduction

Vedolizumab (VDZ) is a humanized monoclonal antibody (mAb) that blocks the interaction of α4β7 integrin and mucosal addressin cell adhesion molecule-1 (MAdCAM-1), thereby inhibiting the migration of memory T cells into inflamed intestinal tissue [1]. According to the European Summary of Product Characteristics, this drug is indicated for the treatment of adults with moderately to severely active ulcerative colitis (UC) or Crohn’s disease (CD). It is reserved for patients who have not responded to, have responded inadequately to, or cannot tolerate conventional therapy (glucocorticosteroids, immunosuppressants) or the tumor necrosis factor-alpha (TNFα) antagonist. The recommended dosing regimen of VDZ is 300 mg administered as an intravenous infusion at 0, 2, and 6 weeks, and every 8 weeks thereafter (Q8W) [2]. In certain cases, an increased dosing frequency of every 4 weeks (Q4W) may be considered for patients who no longer respond to the Q8W regimen [3].

Given the limited number of biologic agents approved for the treatment of UC and CD, therapy optimization becomes paramount. Various factors, such as sex, body size, immunogenicity, albumin levels, and the use of immunosuppressants, have been associated with the accelerated clearance (CL) of mAb, implying wide variability between patients [4]. Thus, therapeutic drug monitoring (TDM) stands out as a crucial tool for the individualization of biologic therapy, whether using a reactive or proactive approach [5]. Reactive TDM of infliximab has become an essential part of managing patients with inflammatory bowel disease (IBD), with growing evidence highlighting the benefits of proactive approach as well [6,7]. For VDZ, TDM is typically recommended at the end of the induction phase in non-responders, and in patients when secondary loss of response is confirmed [5]. However, evidence supporting the routine monitoring of VDZ trough concentrations remains limited, and specific time-dependent and exposure-response targets are still unclear [8,9]. Nevertheless, TDM of VDZ is being utilized more frequently in various clinical settings, even in the absence of robust supporting evidence. Addressing the challenges posed by VDZ pharmacokinetic variability and standardizing TDM practices could provide the basis for establishing universally accepted TDM protocols [9]. In this context, model-informed precision dosing (MIPD) could serve as a valuable tool for personalized biologic therapy. MIPD typically uses a Bayesian approach and population pharmacokinetic models to predict the optimal dosing for achieving a target drug concentration [4]. The method can be used a priori (based on patient covariates) to determine the starting dose, or a posteriori (based on covariates and one or more observed concentrations) to generate a Bayesian posterior parameter distribution, allowing prediction of the next dose [10].

A population pharmacokinetic analysis of VDZ was previously performed in patients with UC or CD using extensively collected data from phase 1 and 2 studies, as well as sparsely collected data from the phase 3 GEMINI studies, in a predominantly Western population. According to this model, only extreme albumin and body weight values were identified as potentially clinically relevant predictors for the linear clearance (CL_L_) of VDZ [11]. Subsequently, other authors have usually adapted this model to specific populations and study conditions [3,12,13,14]. However, the potential use of the developed models in real-world clinical settings remains unknown and requires validation in an independent cohort. Therefore, this study aims to assess the predictive performance of published population pharmacokinetic models of VDZ in patients with IBD to determine their applicability for our population and to subsequently use them in the clinical setting within MIPD. Specifically, the study sought to assess the models’ capacity for a priori predictions using patient covariates only, as well as their utility in simulation-based application. Moreover, the models were utilized for Monte Carlo simulations to generate and compare concentration–time profiles based on clinically important covariate combinations specified within the models.

## 2. Methods

### 2.1. Literature Search

A systematic literature search for published original articles on population pharmacokinetic models for VDZ in patients with IBD until November 2024 was conducted in PubMed, with the language being limited to English. The population studies were searched for using the following terms: (Vedolizumab) AND (pharmacokinetic model OR population pharmacokinetics OR population pharmacokinetic model* OR nonlinear mixed effects model* OR NONMEM OR pharmacometric) AND (Inflammatory Bowel Diseases OR Crohn Disease OR Crohn’s Disease OR ulcerative colitis OR IBD). Moreover, the reference lists of the relevant articles were searched for additional literature. The models were excluded if (1) the study was not a population pharmacokinetic analysis (nonlinear mixed effects modeling analysis) of VDZ in patients with IBD; (2) model parameters were not available for external evaluation; (3) the publications were conference abstracts or review articles; or (4) models were constructed on a population unrepresentative for our population (e.g., pediatric population). The following information was extracted from the articles: patient characteristics, software, pharmacokinetic model structure, typical value of pharmacokinetic parameters, inter-individual variability (IIV), inter-occasion variability (IOV), residual variability (RV), and covariates significantly influencing CL_L_ with potential clinical relevance.

### 2.2. External Evaluation Cohort

We enrolled 106 adult patients diagnosed with UC or CD from the University Medical Center “Zvezdara”, Belgrade, Republic of Serbia, who were treated with VDZ and underwent TDM during the induction or maintenance phase. The study protocol was approved by the institutional Ethics Committee (No. IRB00009457, on 5 October 2022). The design and conduct of this clinical study adhered to the ethical standards outlined in the Declaration of Helsinki. During the induction phase, VDZ (Entyvio^®^, 300 mg vial solution for infusion, Takeda Italia S.P.A., Rome, Italy) was administered as a 30 min intravenous infusion at a standard dose of 300 mg at weeks 0, 2, and 6. Maintenance dosing intervals were 4, 6, or 8 weeks, depending on clinical assessment and the individual patient’s response to treatment.

The required data were extracted from the patients’ medical records, and included VDZ therapy and TDM data (date of therapy initiation, dosing regimen, exact time and date of blood sampling for TDM, and measured concentration), co-therapy (prior and current), demographic (age, sex, body weight), clinical (disease type), and laboratory characteristics including serum albumin concentrations. Patients were excluded from the analysis if data about VDZ dosing and TDM were missing.

VDZ concentration measurements were performed using commercially available R-Biopharm^®^ ELISA tests on a Dynex DS2 analyzer (Dynex Technologies, Chantilly, VA, USA) in the biochemical laboratory of University Hospital Medical Center “Zvezdara”, Belgrade, Republic of Serbia.

### 2.3. External Predictability Evaluation

The external evaluation was conducted using NONMEM version 7.5 (ICON Development Solutions, Ellicott City, MD, USA). The programs R and RStudio were used for the pre- and post-processing of input/output. Published population pharmacokinetic models were coded by inputting the equations and parameter values into the control file and executing with an iteration of 0 (MAXEVAL = 0). Only covariates with potential clinical relevance were considered. Covariates with missing data were assigned the reference values from the selected model. Because no data on anti-drug antibodies (ADAs) were available in the validation dataset, this variable was not considered. Prediction- and simulation-based diagnostics were used to assess the predictive performances of the selected models.

External validation of a model was based on comparison between predictions obtained with the models and VDZ measurements in the external dataset. The predictions represent a priori predictions based on the patients’ covariates with potential clinical relevance. Several metrics used for model evaluation have been previously described [10,15]. The bias and precision of prediction were evaluated using the mean prediction error (MPE, Equation (1)) and root mean square error (RMSE, Equation (2)).

(1) $$\mathrm{MPE}=\frac{\sum \left(\hat{Y}-Y\right)}{n}$$

(2) $$\mathrm{RMSE}=\sqrt{\frac{\sum {\left(\hat{Y}-Y\right)}^{2}}{n}}$$

in which $\hat{Y}$ represents the population-predicted VDZ concentration, Y represents the observed VDZ concentration, and n is the number of observations [16]. Relative prediction errors were expressed as a percentage of the measured value (PE (%)) using Equation (3).

(3) $$\mathrm{PE}\left(\%\right)=\frac{\hat{Y}-Y}{Y}\cdot 100$$

The median prediction error (MDPE (%)), median absolute prediction error (MAPE (%)), and PE (%) values that fell within the range of ±20% (F20) and ±30% (F30) were applied to further evaluate the accuracy and precision of the models, as previously described [17]. To enable a more comprehensive comparison of the models, the results were presented as an overall comparison and as a group comparison. Predictive performance was evaluated across subgroups defined by specific covariates. Moreover, to assess the overall model fit, measured values were plotted against population predictions.

Simulation-based diagnostics were performed using prediction- and variability-corrected visual predictive checks (pvcVPCs). The model of interest was used to generate 1000 simulations of the observed data. The 95% confidence intervals (CI) for the 5th, 50th, and 95th percentiles of the simulations were compared with the prediction- and variability-corrected observations, binning automatically, and plotted against an independent variable. In addition, the normalized prediction distribution error (NPDE) was determined using an add-on R package (NPDE, version 3.5, www.npde.biostat.fr, accessed on 1 October 2024). A total of 1000 datasets were simulated based on the reported final model parameters. A normal distribution N(0, 1) of NPDEs was tested using the Wilcoxon signed-rank test to assess whether the mean value significantly differed from zero and the Fisher variance test to compare the variance to one. Additionally, the Shapiro–Wilk test was conducted to compare the distribution of the NPDE to a normal distribution. Finally, a global test with an adjusted *p*-value of all the three tests was used to identify the best model. To visualize the distribution, quantile–quantile (QQ) plots and histograms of NPDE were presented by the NPDE package in R.

### 2.4. Monte Carlo Simulations Using Selected Models 

Finally, the models were utilized for Monte Carlo simulations to generate concentration–time profiles based on covariate combinations specified within the models and the Q8W dosing regimen. VDZ trough levels in week 22 were compared among subgroups. Specifically, different albumin levels (30 g/L and 40 g/L), body weight (50 kg, 70 kg, and 100 kg), prior biologic therapy, and ADA status were considered, resulting in 24 unique covariate combinations that defined the subgroups. Additionally, VDZ concentrations were simulated (n = 1000) to assess Q8W and Q4W regimens for the best-case (no previous biologic exposure, body weight = 70 kg, no ADA, albumin = 40 g/L) and worst-case (previous biologic exposure, body weight = 100 kg, ADA, albumin = 30 g/L) covariate combinations. For the selected models, the proportion of concentrations in the maintenance phase (week 22) exceeding 12 µg/mL was calculated.

## 3. Results

### 3.1. Review of Selected Population Pharmacokinetic Models

**Figure 1:**
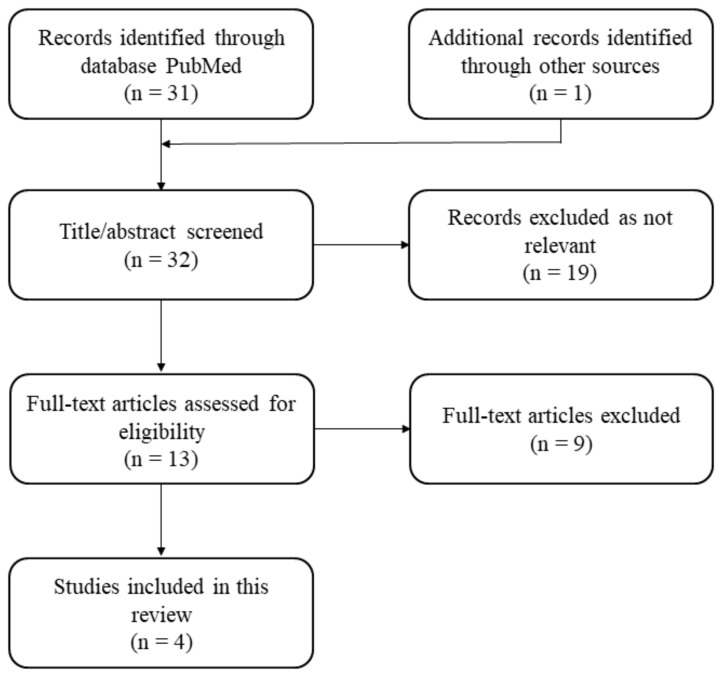
Flow chart of the article selection.

A total of 31 results were found and collected in the PubMed search using the keywords mentioned in Section 2. An independent literature review revealed only one additional result. Following the application of inclusion and exclusion criteria, four population pharmacokinetic models [3,11,13,14] of VDZ in IBD patients were finally selected (Figure 1). The model by Rosario et al. characterized the pharmacokinetics of VDZ using a two-compartment model with parallel linear and nonlinear elimination [11], which served as a reference model. Subsequent studies updated this model to fit their specific datasets. All selected models were developed/updated using NONMEM^®^ software (version 7.5). More detailed information about the selected studies and models can be found in Table 1. In all studies, VDZ was intravenously administered.

Typical values for CL_L_ in the studies ranged from 0.155 to 0.215 L/day, regardless of the disease type [3,11,13,14]. CL_L_ in ADA positive patients was 0.246 L/day in the study by Okamoto et al. [3]. The typical values of V_1_ and V_2_ ranged from 3.16 to 4.92 L, and from 1.65 to 1.84 L, respectively. The reported typical value of K_m_ was 0.851–0.964 µg/mL, while the typical value of V_max_ was 0.238–0.265 mg/day. Three models reported the IIV (percent coefficient of variation) associated with CL_L_ [3,11,14], with values ranging from 26.2% to 34.6%, and two models reported IIV associated with V_1_ of 19.1% [11] and 20.2% [3]. IOV was accounted for by Okamoto et al. (an occasion was defined as a dosing interval with at least one associated pharmacokinetic measurement) with an effect on the CL_L_ of 20.3% [3], and Hanzel et al. (occasion was defined as induction—up to the week 14 dose, or maintenance—from week 14 onward) with an effect on the CL_L_ of 15.2% [14]. The residual variation was described with a proportional model [3,11] or a combined proportional and additive model [14]. Random effects were not reported for the model by Pauwels et al. [13].

Identified covariates with potential clinical relevance for CL_L_ were ADA, serum albumin, body weight, and previous biologic exposure (Table 1). Rosario et al. described several covariates in the population model, but only the effects of albumin and body weight at extreme values on CL_L_ had the potential to be clinically meaningful (effect sizes greater than ±25%) [11]. Thus, only these two covariates were accounted in the external evaluation analysis. On the other hand, the effects of fecal calprotectin (FCP), Crohn’s Disease Activity Index (CDAI) score, partial Mayo score, age, prior TNF-α antagonist therapy status, ADA status (ELISA assay), and concomitant therapy use on VDZ CL_L_ were not considered clinically important in the Rosario et al. model [11], and Bayesian 95% credible intervals 95% (CDIs) for most effects contained the null effect. In the subsequent analysis, the remaining effects of sex and CRP were not clinically important. Only graphical evaluation implied that at week 6, UC patients with a Mayo endoscopic sub-score of 3 had, on average, 25% higher CL_L_ than patients with a sub-score of 0 [11].

Okamoto et al. updated the reference model in order to assess the impact of covariates on its pharmacokinetics in Asian and non-Asian UC and CD patients. This analysis had updated electrochemiluminescence (ECL) assay data and time-varying ADA in the dataset, so continuous titer data could be used for modeling the ADA effect. Albumin, body weight, and ADA were assigned as clinically meaningful predictors of VDZ CL_L_, while the effect of race and diagnosis on CL_L_ was negligible [3]. The external evaluation analysis included albumin and body weight as covariates, as ADA values were not available for this analysis.

Pauwels et al. reanalyzed the reference model with two compartments, incorporating a third compartment to account for intestinal tissue concentrations, thereby developing a serum–tissue pharmacokinetic model. In the serum pharmacokinetic model, the negative impact of albumin on CL_L_ was incorporated, while body weight data were not available and therefore not considered [13].

Hanzel et al. developed a new VDZ model, building upon a previously published two-compartment model with parallel linear and nonlinear elimination. Their results demonstrated that CL_L_ was higher in patients with lower serum albumin concentrations, in the presence of ADA, and in patients with prior exposure to other biologic therapy [14]. Consequently, these covariates were considered in the external evaluation analysis.

### 3.2. Characteristics of External Cohort

Our cohort for external evaluation included data from 62 patients with UC and 44 patients with CD. The characteristics of patients and therapy are given in Table 2. A total of 114 VDZ trough concentration measurements were obtained during the follow-up period.

### 3.3. Prediction-Based Diagnostics

**Figure 2:**
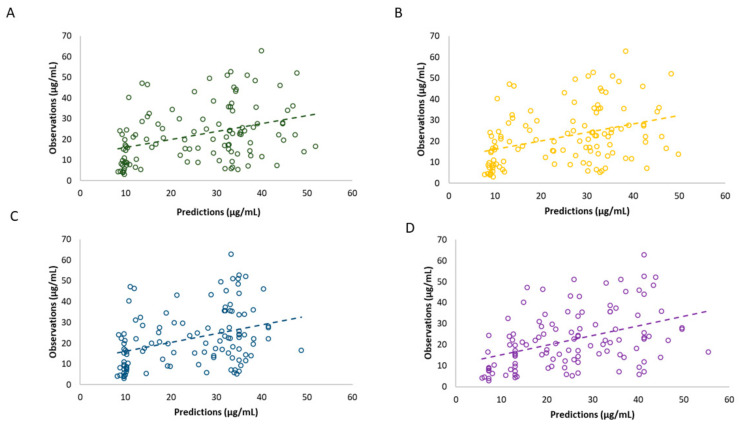
Observed vedolizumab concentrations vs. population predictions of the evaluated models: (**A**) Rosario et al., 2015 [11], (**B**) Okamoto et al., 2021 [3], (**C**) Pauwels et al., 2021 [13], and (**D**) Hanzel et al., 2022 [14]. Values are shown as points with a dashed regression line through the data.

**Figure 3:**
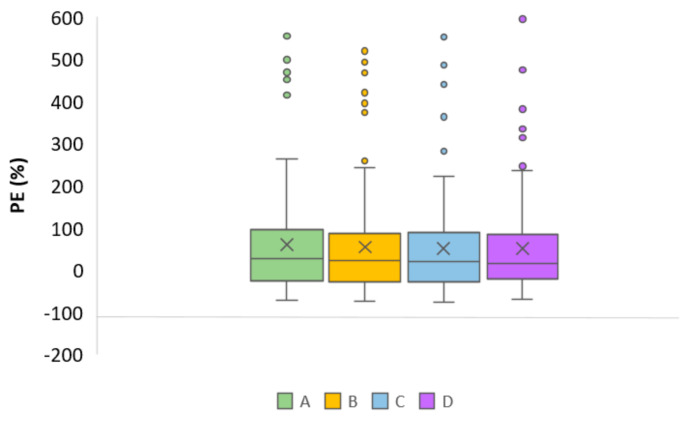
Boxplot for prediction error (PE (%)) of the evaluated models: (A) Rosario et al., 2015 [11], (B) Okamoto et al., 2021 [3], (C) Pauwels et al., 2021 [13], and (D) Hanzel et al., 2022 [14]. The × represents the mean value, the central line within each box represents the median, while the edges of the box denote the interquartile range (IQR); the vertical lines are whiskers and individual points are outliers.

The prediction-based diagnostic results are summarized in Table 3, while the population-predicted values vs. measured values are presented in Figure 2 and relative PE (%) in the boxplot in Figure 3. Although none of the models met the combined criteria of MDPE ≤ ±20%, MAPE ≤ 30%, F20 ≥ 35%, and F30 ≥ 50% (Table 3), the models proposed by Pauwels et al. and Hanzel et al. exhibited lower MDPE (≤20%) compared to the other two models, demonstrating superior accuracy. However, the CI of MPE included 0 only for the model by Pauwels et al. [13]. In terms of precision, the model of Hanzel et al. [14] showed the lowest MAPE of 41.64%, but, in general, all models exceeded the acceptable limit of 30%.

The prediction-based performance was evaluated across subgroups defined by specific covariate. All of the models performed better in terms of accuracy and precision for the subgroup with low–normal albumin levels, while subgroups with different body weights (≤70 kg and >70 kg) showed variable results. The Hanzel et al. [14] model showed superior characteristics for the subgroup of patients with prior biologic exposure. However, none of the models showed compliance with the combined criteria.

### 3.4. Simulation-Based Diagnostics

**Figure 4:**
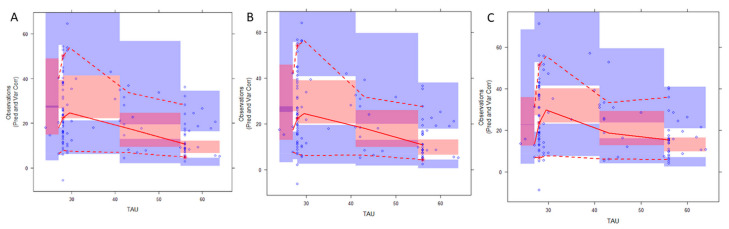
Prediction- and variability-corrected visual predictive check (pvcVPC) of the evaluated models: (**A**) Rosario et al., 2015 [11], (**B**) Okamoto et al., 2021 [3], and (**C**) Hanzel et al., 2022 [14]; prediction- and variability-corrected concentrations (µg/mL) vs. time after dose (days). The open black circles represent the observed vedolizumab (VDZ) concentrations. Solid and dashed red lines represent the median, 5th, and 95th percentiles of the observed data. The semi-transparent red shaded area represents the simulation-based 95% confidence interval (CI) for the median, while the semi-transparent blue fields represent the 95% CI around the 5th and 95th percentiles of the simulation-based prediction intervals.

In the simulation-based diagnostics, the pvcVPC was used for diagnosis if the model accurately predicted both the central trend and the variability of the data (Figure 4). The results showed that the 5th and 95th (90%) percentile PIs covered most of the observations for each model. Although performance of the models was similar, slight deviations may be observed between observed and simulated data for each model, with the most obvious deviation at the 5th percentiles of the Hanzel et al. model [14].

**Figure 5:**
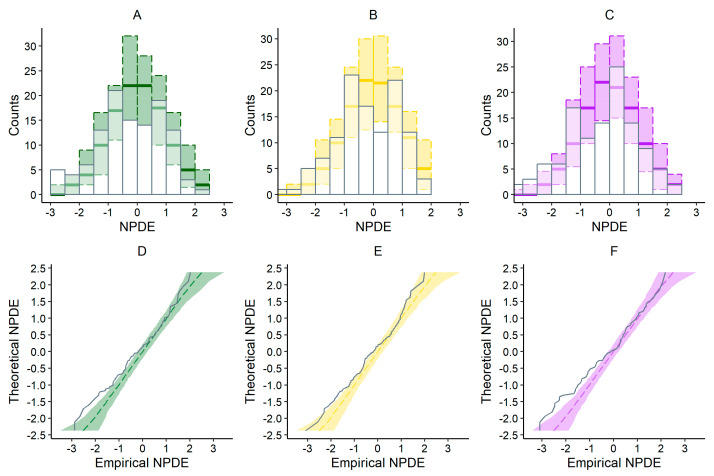
Plots illustrating normalized prediction distribution errors (NPDEs) of the evaluated models; histogram of NPDE of models: (**A**) Rosario et al., 2015 [11], (**B**) Okamoto et al., 2021 [3], and (**C**) Hanzel et al., 2022 [14]; QQ plot of theoretical NPDE vs. empirical NPDE of models (**D**) Rosario et al., 2015 [11], (**E**) Okamoto et al., 2021 [3], and (**F**) Hanzel et al., 2022 [14].

Plots illustrating the NPDE of the evaluated models are presented on Figure 5, and results of statistical analysis are presented in Table 4. Statistical analysis confirmed normal distribution of NPDE N(0,1) for the Rosario et al. [11] and Okamoto et al. [3] models (Table 4). Even though the majority of NPDE values for the Hanzel et al. [14] model were within the range of plus or minus 1.96, variance was significantly different from one (*p* < 0.01).

### 3.5. Simulated VDZ Concentration-Time Profiles

**Figure 6:**
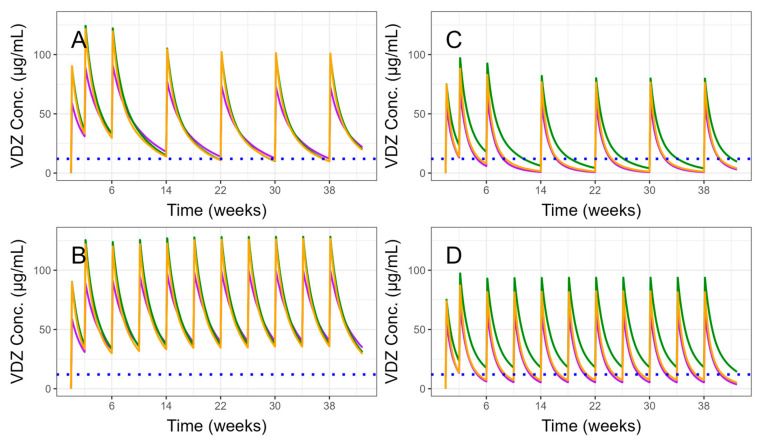
Simulated median vedolizumab (VDZ) pharmacokinetic profiles for the best-case: no previous biologic exposure, body weight = 70 kg, no ADA, albumin = 40 g/L (**A**,**B**) and the worst-case: previous biologic exposure, body weight = 100 kg, ADA, albumin = 30 g/L combination of covariates (**C**,**D**) following dosing frequency of 8 weeks (**A**,**C**) and 4 weeks (**B**,**D**) in the maintenance phase using three population pharmacokinetic models. Purple—Hanzel et al., 2022 [14], yellow—Okamoto et al. [3], green—Rosario et al. [11]. Blue dotted line represents y = 12 µg/mL.

Simulated VDZ concentration–time profiles for 24 distinct subgroups, defined by combinations of covariates, following the recommended dosing regimen, are presented in Figures S1–S3 for the three evaluated models. Median concentrations based on 1000 simulations for 8-week and 4-week dosing frequencies in the maintenance phase are shown in Figure 6, comparing the best-case (no previous biologic therapy, body weight = 70 kg, no ADA, albumin = 40 g/L) and the worst-case (previous biologic therapy, body weight = 100 kg, ADA, albumin = 30 g/L) scenarios. Table 5 displays median simulated VDZ trough concentrations at week 22 after 300 mg Q8W in the maintenance phase for different covariate combinations across various models. Additionally, Table 6 summarizes the percentage of virtual patients whose VDZ troughs exceeded 12 µg/mL in week 22 for different dosing scenarios.

## 4. Discussion

The individualization of biologic exposure is challenging due to the substantial within- and between-subject variability in CL, which may be better predicted using population pharmacokinetic models. Although several population pharmacokinetic studies of VDZ are available for adult IBD patients, the predictability of models has not been assessed on an independent population. To the best of our knowledge, this study is the first to evaluate the published population models using an external dataset. In our study, a validation dataset consisting of 114 VDZ concentrations obtained from 106 IBD patients was used to explore the predictive performance of four population models for VDZ in adults. The evaluation was performed using prediction-based and simulation-based diagnostics.

Previous population analyses characterized the pharmacokinetics of VDZ by a two-compartment model with a nonlinear elimination mechanism at low concentrations and a linear pathway at higher, therapeutic concentrations (Table 1). The nonlinear elimination is related to saturable, target-mediated mechanisms such as receptor-mediated endocytosis, while the linear pathway involves non-saturable components such as Fc-mediated elimination [1,11]. According to the selected models, clinically relevant change in CL_L_ can be expected with extreme values of (low) albumin and (high) body weight, ADA, and previous biologic therapy. However, a portion of the variability in CL_L_ (26.2–34.6%) remains unexplained, suggesting that factors influencing VDZ pharmacokinetics have not yet been definitively established.

In our external evaluation analysis, none of the models met the combined criteria of MDPE ≤ ±20%, MAPE ≤ 30%, F20 ≥ 35%, and F30 ≥ 50% (Table 3). Among the evaluated models, the MDPE (%) results indicated that the predictions from the models by Pauwels et al. and Hanzel et al. demonstrated acceptable accuracy. However, based on MPE results, only the CI for the Pauwels et al. model included 0. Due to the significant skewness and variability observed in the MPE data for each model, MDPE likely provides a more reliable representation of the overall trend. Regarding the precision, the Hanzel et al. model showed the best MAPE of 41.64%, but in general all models exceeded the acceptable limit of 30%. Consistently, the evaluated models displayed similar results in overall model fit, with notable dispersion of population predictions (Figure 2). Interestingly, all models provided better accuracy and precision for the subgroup of patients for whom albumin levels were low–normal (approximately below 40 g/L), but in general none fulfilled the combined criteria. Perhaps a more heterogenous dataset is required to assess predictive performance in covariate-defined subgroups. Overall, all models exhibited bias in population predictions, indicating limited utility for a priori dose adjustments based solely on population parameters and identified covariates. Significant bias in population predictions has also been reported for other monoclonal antibodies used in IBD treatment, such as infliximab [18]. Nevertheless, Bayesian forecasting is expected to improve the precision and accuracy of predictions by incorporating prior concentration data, warranting further evaluation of population models combined with Bayesian estimation. Indeed, for drugs with fixed initial dosing regimens, a posteriori predictions are generally more valuable for optimizing dosing strategies. However, in the absence of TDM data for a posteriori MIPD, predictions based solely on patient covariates may still serve as a useful tool to identify individuals at higher risk of inadequate response. Despite their poor performance in population-based predictions, three tested models showed better results in simulation-based diagnostics performed by pvcVPC (Figure 4). While slight deviations were noted between observed and simulated data for each model, the results suggest that these models are better suited for exploring “what if” scenarios through simulations than prediction-based applications [19]. This divergence between prediction-based and simulation-based evaluations has also been noted in studies involving other therapeutic agents [20,21]. However, additional analysis of NPDE (Figure 5, Table 4) revealed that only the models by Rosario et al. [11] and Okamoto et al. [3] completely fulfilled the criteria. While the majority of NPDEs were within the range of plus or minus 1.96, the variance was significantly different from one (*p* < 0.01) for the model of Hanzel et al [14].

In general, the selected models showed poor population prediction, likely influenced by multiple factors. The extrapolation may have been limited by missing some clinically relevant covariates in the evaluation dataset, such as ADA or albumin values in some patients. This limitation may be a potential reason for the detection of several outliers presented in Figure 3. Furthermore, the variables contributing to VDZ pharmacokinetic variability have not been definitively established. Although several covariates were described previously, only albumin, body weight, ADA, and previous biologic therapy showed a clinically relevant change in CL_L_ in selected population models. Consequently, the external validation of these models may have been hindered by their inability to account for all covariates relevant to the external population.

Albumin levels were consistently found to be negatively associated with CL_L_ across all evaluated VDZ models, aligning with previous findings [4]. While the exact mechanism underlying this effect is not fully understood, it has been proposed that reduced expression or activity of the neonatal Fc receptor (FcRn) in IBD patients impairs salvage of albumin and IgG, reflecting increased antibody CL. Another possible mechanism is that inflammation of the gastrointestinal tract in IBD patients may lead to an atypical elimination route [11]. Both Rosario et al. [11] and Okamoto et al. [3] emphasized that only the extremely low albumin levels were identified as potentially clinically important predictors of CL_L_. Although none of the patients in the study by Pauwels et al. [13] had low albumin levels (<32 g/L), this covariate was incorporated into the model to confirm previous findings [13]. Hanzel et al. [14] calculated that CL_L_ increased at lower albumin levels (28 g/L) for 26% compared to a typical patient [14]. Indeed, the clinical relevance of this covariate for VDZ pharmacokinetics was confirmed in other studies as well [12].

The disposition of monoclonal antibodies, including VDZ, is often described as a function of body size [4,22]. The models by Rosario et al. [11] and Okamoto et al. [3] incorporated body weight using allometric scaling. Allometric power coefficients were fixed to 0.75 for V_max_ and Q, and to 1.0 for V_2_, while the weight effect was estimated for V_1_ and CL_L_. Both studies found that only extreme body weight values were identified as potentially clinically important predictors of CL_L_ [3,11]. Rosario et al. [11] noted that a typical individual weighing 120 kg had a 19% probability of having a CL_L_ value more than 25% higher than in a patient weighing 70 kg [11]. In contrast, Hanzel et al. [14] did not find an effect of body weight on VDZ pharmacokinetics, likely due to the narrower body weight range in their cohort [14]. In addition to body size, other demographic variables, such as age, sex, and race, were also evaluated as potential covariates. Okamoto et al. [3] studied the pharmacokinetics of VDZ in Asian and non-Asian patients, finding only a small, clinically insignificant effect of race [3]. Similarly, age and sex were determined not to be a significant or clinically relevant covariates for VDZ CL_L_ in selected studies [3,11,14].

As exogenous proteins, therapeutic monoclonal antibodies can provoke an immune response, leading to the production of ADA, which can accelerate the elimination of biologics [4]. In the study by Rosario et al., the presence of ADAs (either persistently or transiently) was found to increase VDZ CL_L_ by only 12%, a statistically significant but clinically insignificant effect [11]. In contrast, Okamoto et al. [3] identified a positive ADA titer as a potentially clinically important predictor of CL_L_. Specifically, CL_L_ increased from 0.165 in ADA-negative patients (<10 titers) to 0.246 L/day in ADA-positive patients (titer 250) [3]. Moreover, Hanzel et al. [14] observed a marked increase in CL_L_ by 89% in the presence of ADAs [14]. Simulated concentration–time profiles across various covariate subgroups revealed a dominant effect of ADA on CL_L_, particularly for the models developed by Hanzel et al. [14] and Okamoto et al. [3] (Figures S1 and S2). These findings highlight the significance of ADA as a covariate affecting VDZ pharmacokinetics and underscore the potential for immunogenicity to influence therapeutic outcomes in clinical settings.

Monoclonal antibody interactions with small-molecule drugs are generally not anticipated [22]. However, prior biologic therapy has occasionally been associated with higher CL of monoclonal antibodies in IBD patients [4]. Hanzel et al. found that CL_L_ increased by 25% in patients who had prior exposure to biologics compared to those with no prior exposure [14]. This impact of prior biologic therapy on clearance was reflected in the simulated concentrations (Figure S1). On the other hand, Rosario et al. [11] detected a minor 4% increase in CL_L_ in patients who had been treated with and failed prior TNF-α antagonist therapy, although this effect was not deemed clinically meaningful. Moreover, immunomodulatory therapies, or amino-salicylate or corticosteroid therapy, were not detected as being important for the pharmacokinetic variability of VDZ [11,14].

There was no clinically meaningful effect of IBD diagnosis on CL_L_ in the model by Okamoto et al. [3]. Likewise, typical estimates of CL_L_ were similar between UC and CD patients within the Rosario et al. model [11]. The effect of the partial Mayo score and the CDAI score at baseline had no effect on VDZ CL_L_ in Rosario et al. model [11]. However, a trend toward lower CL_L_, and therefore higher concentrations, in UC patients with a lower Mayo endoscopic sub-score was noted. As additional measures of disease severity, the effects of FCP and CRP concentrations on VDZ CL_L_ were evaluated. While the effect of FCP was statistically significant, it was small and not considered to be clinically relevant. Similarly, subsequent post hoc analysis suggested that the effect of CRP on VDZ CL_L_ was also not clinically relevant [11]. Hanzel et al. tested CDAI, CRP, and Simple Endoscopic Score for Crohn’s Disease (SES-CD) at baseline as covariates, but no effect was detected [14].

During the standard induction phase, VDZ trough levels are typically around 27 µg/mL. In the maintenance phase, mean steady-state trough levels are approximately 12 µg/mL for a dosing frequency of Q8W and 36 µg/mL for a dosing frequency of Q4W [2,23,24,25]. In general, VDZ trough concentration >20 μg/mL at week 6 and >12 μg/mL during maintenance are proposed thresholds [26,27,28]. Our simulation analysis reveals that the median concentration is above 12 μg/mL in week 22 during maintenance for approximately 45–60% patients with best-case combinations of covariates and the dosing frequency Q8W (Table 6). Simulated concentrations are notably lower for worst-case combinations of covariates (Table 5, Figure 6), which implies that alternative dosing could be more appropriate. However, simulation analysis with intensified dosing showed variable results. While simulation based on the model by Rosario et al. [11] predicts approximately 77% of the concentration above the threshold, the two other models with an incorporated ADA influence predict notably lower percentages (Table 6, Figure 6). Thus, in patients with ADA-negative status, the Q4W dosing regimen might be appropriate. On the other hand, sub-therapeutic concentrations are likely in ADA-positive patients, especially when additional significant covariates are present (Figures S1 and S2). Moreover, at low VDZ concentrations, nonlinear elimination becomes predominant, further contributing to an increase in CL. Thus, alternative biologic therapy, rather than a pharmacokinetic-guided dosing regimen, might be more suitable for these patients. Yet, it should be noted that VDZ is a humanized monoclonal antibody and its immunogenicity is generally low [12].

Our study has several limitations. As previously mentioned, due to the retrospective nature of data collection from routine TDM, albumin levels were not available for every patient, and ADAs are not part of routine monitoring as VDZ immunogenicity is generally low. Consequently, in subgroup analysis, missing data on albumin were categorized as a low–normal level. On the other hand, it is expected that Bayesian forecasting could enhance predictions by utilizing previous concentration points. Unfortunately, this approach could not be applied in our analysis as most patients had only a single VDZ concentration measurement available. Lastly, due to the small number of published VDZ population pharmacokinetic models, we included articles with overlapping datasets but excluded those that were missing key pharmacokinetic parameters. These limitations may have impacted the generalizability and accuracy of the results, and future studies with more comprehensive datasets are necessary to validate and improve model predictions.

## 5. Conclusions

We evaluated four published VDZ population pharmacokinetic models for patients with IBD using an independent dataset from our center. The external predictive performance of these models was not satisfactory in prediction-based diagnostics, which indicates that a priori predictions based solely on covariates remains challenging. However, combining model-based dosing with therapeutic drug monitoring (TDM) could offer a more reliable approach for a posteriori predictions, and this should be further explored in future studies. When drug concentration measurements are available, Bayesian estimation enables the derivation of individual pharmacokinetic parameters, allowing the optimization of dosing strategies tailored to each patient. Our findings suggest that two of the tested models may, in particular, be more suitable for simulation-based applications. The simulations conducted using these models contribute valuable insights into optimizing the VDZ dosing regimen for patients with varying characteristics. These results emphasize the potential utility of simulation-based approaches in tailoring treatment for individual patients, although further validation is required to fully assess their clinical applicability.

## Figures and Tables

**Table 1:** Summary of selected (developed/updated) population pharmacokinetic studies of vedolizumab (VDZ) and identified factors of variability in linear clearance with potential clinical relevance.

Reference	Population Number of Subjects (Disease Type); Age	Software/Model	Typical Value of Pharmacokinetic Parameters, IIV, IOV and RV	Covariates Significantly Influenced CL_L_ with Potential Clinical Relevance
Developed population pharmacokinetic models				
Rosario et al., 2015 [11]	2554 individuals: 87 healthy volunteers from phase 1 study, 46 patients from phase 2 study (UC), and 891, 1115, and 415 patients from phase 3 GEMINI 1 (UC), GEMINI 2 (CD), and GEMINI 3 (CD) studies; age median (range): 36 (18–78) years	NONMEM/ 2-COMP model with parallel linear and nonlinear elimination	CL_L_: 0.159 L/day (UC) CL_L_: 0.155 L/day (CD) K_m_: 0.964 µg/mL V_max_: 0.265 mg/day Q: 0.12 L/day V_1_: 3.19 L V_2_: 1.65 L IIV CL_L_: 34.6%, corrCL_L_–V_1_: 0.566, IIV V_1_: 19.1%, corrCL_L_–V_max_: −0.192, corrV_1_–V_max_: −0.267, IIV V_max_: 105% Proportional error: 0.0554 (23.5%)	ALB, WT
Population pharmacokinetic model reanalysis/update of previous model				
Okamoto et al., 2020 [3]	1933 individuals: 9 patients from phase 1 study (CPH-001, UC), 743, 966, 152, and 63 patients from phase 3 GEMINI 1 (UC), GEMINI 2 (CD), CCT-101 (UC), and CCT-001 (CD) studies; age median (range): 36 (17–79) years	NONMEM/ 2-COMP model with parallel linear and nonlinear elimination	CL_L_: 0.165 L/day (ADA−) 0.246 L/day (ADA+) K_m_: 0.851 μg/mL V_max_: 0.238 mg/day Q: 0.161 L/day V_1_: 3.16 L V_2_: 1.84 L IIV CL_L_: 30.8% corr CL_L_–V_1_: 0.581 IIV V_1_: 20.2% corr CL_L_–V_2_: 0.0188 corr V_1_-V_2_: 0.371 IIV V_2_: 70.2% IOV CL_L_: 20.3% Proportional error: 0.0318 (17.8%)	WT, ALB, ADA status and titer
Pauwels et al., 2021 [13]	37 patients: 12 (UC), 3 (IBD-U), 22 (CD); age median (IQR): 39 (26–50) years	NONMEM/ Serum PK model: 2-COMP model with parallel linear and nonlinear elimination Serum-tissue PK model: 3-comp model	Serum PK model: CL_L_: 0.159 L/day (UC), 0.155 L/day (CD) K_m_: 0.964 mg/L V_max_: 0.265 mg/day Q: 0.12 L/day V_1_: 3.19 L V_2_: 1.65 L Serum—tissue PK model: Q_2_: 0.07 L/day V_3_: 1.31 L	ALB
Hanzel et al., 2022 [14]	108 patients from LOVE-CD trial; age median (IQR): 36 (28–46) years	NONMEM/ 2-COMP model with parallel linear and nonlinear elimination	CL_L_: 0.215 L/day K_m_: 0.964 mg/L fixed V_max_: 0.265 mg/day fixed Q: 0.12 L/day fixed V_1_: 4.92 L V_2_: 1.65 L fixed IIV CL_L_: 26.2% IOV CL_L_: 15.2% Additive error: 0.469 mg/L Proportional error: 18.9%	ALB, ADA status, no previous biologic exposure

IIV—inter-individual variability in %; IOV—inter-occasional variability in %; RV—residual variability; 2-COMP—2-compartment model; IQR—interquartile range; COMP—compartment; PK—pharmacokinetic; CL_L_—clearance of linear elimination pathway; K_m_—concentration at half-maximum elimination rate; V_max_—maximum elimination rate; V_1_—apparent volume of the central compartment; V_2_—apparent volume of the peripheral compartment; V_3_—apparent volume of the third compartment; Q—apparent intercompartmental clearance between first and second compartment; Q_2_—apparent intercompartmental clearance between second and third compartment; corr—correlation coefficient; ALB—albumin; ADA—anti-drug antibody; WT—body weight.

**Table 2:** Patient and therapy characteristics of the external evaluation dataset.

Characteristic (Units)	n (%)/Mean ± SD (Range)
Diagnosis	
UC	62 (58.49%)
CD	44 (41.51%)
Age (years)	49.76 ± 17.43 (21–78)
Sex (male)	55 (51.9%)
Body weight (kg)	71.94 ± 12.76 (44–110)
Albumin (g/L) *	42.93 ± 4.63 (30–55)
Immunomodulatory drug	86 (81.1%)
Prior anti-TNFα therapy	44 (41.5%)
Vedolizumab concentrations (µg/mL)	
TAD 4 weeks	26.09 ± 14.30 (5.309–62.79)
TAD 6 weeks	23.43 ± 8.53 (9.119–43.08)
TAD 8 weeks	15.39 ± 11.40 (3.02–47.22)

n—number; SD—standard deviation; UC—ulcerative colitis; CD—Crohn’s disease; TAD—time after dose; * Missing data in 62 patients (54.4%).

**Table 3:** Comparison of population (PRED) predictions to observations for the evaluated population models of vedolizumab (VDZ).

Population PK Model	MPE (µg/mL) 95% CI	MDPE (%)	RMSPE (µg/mL)	F20 (%)	F30 (%)	MAPE (%)
Rosario et al., 2015 [11]	3.77 1.05–6.50	25.57	15.25	20.18	29.82	47.56
Okamoto et al., 2021 [3]	2.84 0.156–5.53	20.75	14.85	21.05	28.95	44.58
Pauwels et al., 2021 [13]	2.06 −0.567–4.68	18.63	14.38	21.93	28.07	45.96
Hanzel et al., 2022 [14]	2.91 0.331–5.49	13.82	14.30	25.44	38.60	41.64

MPE—mean prediction error, CI—confidence interval, MDPE—median prediction error, RMSPE—root mean square prediction error; F20—percentages of PE% within ±20%, F30—percentages of PE% within ±30%, MAPE—median absolute prediction error.

**Table 4:** Statistical tests for evaluating the normality of the normalized prediction distribution errors (NPDEs).

Population PK Model	Wilcoxon *	Fisher *	Shapiro–Wilks *	Global *
Rosario et al., 2015 [11]	0.0958	0.155	0.381	0.0958
Okamoto et al., 2021 [3]	0.132	1	0.578	0.132
Hanzel et al., 2022 [14]	0.0802	0.00894	0.394	0.00894

* data are expressed as *p*-values.

**Table 5:** Simulated vedolizumab trough concentration in week 22 after 300 mg every 8 weeks in the maintenance treatment based on a combination of covariates using different population pharmacokinetic models.

Population PK Model	Median Trough Concentration (IQR) of Vedolizumab (VDZ) in Week 22 [µg/mL]	
	Best-Case Combination of Covariates ^1^	Worst-Case Combination of Covariates ^2^
Hanzel et al. [14]	13.83 (3.62–34.46)	0.77 (0.12–4.61)
Okamoto et al. [3]	10.86 (1.54–35.89)	1.32 (0.03–8.20)
Rosario et al. [11] *	10.98 (1.74–37.19)	4.31 (0.36–18.86)

^1^ no previous biologic exposure = 70 kg, no ADA, albumin = 40 g/L; ^2^ previous biologic exposure, body weight = 100 kg, ADA, albumin = 30 g/L; * for Crohn’s disease indication.

**Table 6:** Percent of simulated patients with vedolizumab (VDZ) trough concentration above 12 µg/mL in week 22 using different population pharmacokinetic (PK) models.

Population PK Model	Patients with Trough Concentration of Vedolizumab (VDZ) >12 µg/mL in Week 22 [%]	
	Best-Case Combination of Covariates ^1^ and 300 mg Every 8 Weeks During Maintenance	Worst-Case Combination of Covariates ^2^ and 300 mg Every 4 Weeks During Maintenance
Hanzel et al. [14]	60.8	9.5
Okamoto et al. [3]	44.1	22.6
Rosario et al. [11] *	45.5	77.3

^1^ no previous biologic exposure, body weight = 70 kg, no ADA, albumin = 40 g/L; ^2^ previous biologic exposure, body weight = 100 kg, ADA, albumin = 30 g/L; * for Crohn’s disease indication.

## Data Availability

The data presented in this study are available on request from the corresponding author due to privacy and ethical reasons.

## Supplementary Materials

The following supporting information can be downloaded at: https://www.mdpi.com/article/10.3390/biomedicines13010043/s1, Figure S1: Simulated vedolizumab (VDZ) concentrations with various combinations of covariates (ALB = albumin level, ADA = anti-drug antibody status, BW = body weight, ATNF naive = no previous biologic therapy) for dosing regimen (300 mg, dosing frequency Q8W) for the model by Hanzel et al., 2022 [14]; Figure S2: Simulated vedolizumab (VDZ) concentrations with various combinations of covariates (ALB = albumin level, ADA = anti-drug antibody status, BW = body weight, ATNF naive = no previous biologic therapy) for dosing regimen (300 mg, dosing frequency Q8W) for the model by Okamoto et al., 2020 [3]; Figure S3: Simulated vedolizumab (VDZ) concentrations with various combinations of covariates (ALB = albumin level, ADA = anti-drug antibody status, BW = body weight, ATNF naive = no previous biologic therapy) for dosing regimen (300 mg, dosing frequency Q8W) for the model by Rosario et al., 2015 [11].

## References

[1] Rosario M., Dirks N.L., Milch C., Parikh A., Bargfrede M., Wyant T., Fedyk E., Fox I. (2017). A review of the clinical pharmacokinetics, pharmacodynamics, and immunogenicity of vedolizumab. Clin. Pharmacokinet..

[2] SmPC Entyvio Summary of Product Characteristics for Entyvio 300 mg Powder for Concentrate for Solution for Infusion. https://www.medicines.org.uk/emc/product/5442/smpc#gref.

[3] Okamoto H., Dirks N.L., Rosario M., Hori T., Hibi T. (2021). Population pharmacokinetics of vedolizumab in Asian and non-Asian patients with ulcerative colitis and Crohn’s disease. Intest. Res..

[4] Deyhim T., Cheifetz A.S., Papamichael K. (2023). Drug clearance in patients with inflammatory bowel disease treated with biologics. J. Clin. Med..

[5] Papamichael K., Cheifetz A.S., Melmed G.Y., Irving P.M., Vande Casteele N., Kozuch P.L., Raffals L.E., Baidoo L., Bressler B., Devlin S.M. (2019). Appropriate therapeutic drug monitoring of biologic agents for patients with inflammatory bowel diseases. Clin. Gastroenterol. Hepatol..

[6] Sethi S., Dias S., Kumar A., Blackwell J., Brookes M.J., Segal J.P. (2023). Meta-analysis: The efficacy of therapeutic drug monitoring of anti-TNF-therapy in inflammatory bowel disease. Aliment. Pharmacol. Ther..

[7] Cheifetz A.S., Abreu M.T., Afif W., Cross R.K., Dubinsky M.C., Loftus E.V., Osterman M.T., Saroufim A., Siegel C.A., Yarur A.J. (2021). A comprehensive literature review and expert consensus statement on therapeutic drug monitoring of biologics in inflammatory bowel disease. Am. J. Gastroenterol..

[8] Dreesen E., Verstockt B., Bian S., de Bruyn M., Compernolle G., Tops S., Noman M., Van Assche G., Ferrante M., Gils A. (2018). Evidence to support monitoring of vedolizumab trough concentrations in patients with inflammatory bowel diseases. Clin. Gastroenterol. Hepatol..

[9] Di Paolo A., Luci G. (2020). Personalized medicine of monoclonal antibodies in inflammatory bowel disease: Pharmacogenetics, therapeutic drug monitoring, and beyond. Front. Pharmacol..

[10] El Hassani M., Marsot A. (2023). External evaluation of population pharmacokinetic models for precision dosing: Current state and knowledge gaps. Clin. Pharmacokinet..

[11] Rosario M., Dirks N.L., Gastonguay M.R., Fasanmade A.A., Wyant T., Parikh A., Sandborn W.J., Feagan B.G., Reinisch W., Fox I. (2015). Population pharmacokinetics-pharmacodynamics of vedolizumab in patients with ulcerative colitis and Crohn’s disease. Aliment. Pharmacol. Ther..

[12] Wyant T., Yang L., Rosario M. (2020). Comparison of the ELISA and ECL assay for vedolizumab anti-drug antibodies: Assessing the impact on pharmacokinetics and safety outcomes of the phase 3 GEMINI trials. AAPS J..

[13] Pauwels R.W.M., Proietti E., van der Woude C.J., Oudijk L., Crombag M.B.S., Peppelenbosch M.P., Grohmann U., Fuhler G.M., de Vries A.C. (2021). Vedolizumab tissue concentration correlates to mucosal inflammation and objective treatment response in inflammatory bowel disease. Inflamm. Bowel Dis..

[14] Hanzel J., Dreesen E., Vermeire S., Lowenberg M., Hoentjen F., Bossuyt P., Clasquin E., Baert F.J., D’Haens G.R., Mathot R. (2022). Pharmacokinetic-pharmacodynamic model of vedolizumab for targeting endoscopic remission in patients with Crohn disease: Posthoc analysis of the LOVE-CD study. Inflamm. Bowel Dis..

[15] Brendel K., Dartois C., Comets E., Lemenuel-Diot A., Laveille C., Tranchand B., Girard P., Laffont C.M., Mentre F. (2007). Are population pharmacokinetic and/or pharmacodynamic models adequately evaluated? A survey of the literature from 2002 to 2004. Clin. Pharmacokinet..

[16] Sheiner L.B., Beal S.L. (1981). Some suggestions for measuring predictive performance. J. Pharmacokinet. Biopharm..

[17] Chen S., Huang L., Huang W., Zheng Y., Shen L., Liu M., Chen W., Wu X. (2024). External evaluation of population pharmacokinetic models for high-dose methotrexate in adult patients with hematological tumors. J. Clin. Pharmacol..

[18] Konecki C., Feliu C., Cazaubon Y., Giusti D., Tonye-Libyh M., Brixi H., Cadiot G., Biron A., Djerada Z. (2021). External evaluation of population pharmacokinetic models and bayes-based dosing of infliximab. Pharmaceutics.

[19] Cheng Y., Wang C.Y., Li Z.R., Pan Y., Liu M.B., Jiao Z. (2021). Can population pharmacokinetics of antibiotics be extrapolated? Implications of external evaluations. Clin. Pharmacokinet..

[20] Ryu S., Jung W.J., Jiao Z., Chae J.W., Yun H.Y. (2021). External evaluation of the predictive performance of seven population pharmacokinetic models for phenobarbital in neonates. Br. J. Clin. Pharmacol..

[21] Zhao C.Y., Jiao Z., Mao J.J., Qiu X.Y. (2016). External evaluation of published population pharmacokinetic models of tacrolimus in adult renal transplant recipients. Br. J. Clin. Pharmacol..

[22] Dirks N.L., Meibohm B. (2010). Population pharmacokinetics of therapeutic monoclonal antibodies. Clin. Pharmacokinet..

[23] Feagan B.G., Rutgeerts P., Sands B.E., Hanauer S., Colombel J.F., Sandborn W.J., Van Assche G., Axler J., Kim H.J., Danese S. (2013). Vedolizumab as induction and maintenance therapy for ulcerative colitis. N. Engl. J. Med..

[24] Sandborn W.J., Feagan B.G., Rutgeerts P., Hanauer S., Colombel J.F., Sands B.E., Lukas M., Fedorak R.N., Lee S., Bressler B. (2013). Vedolizumab as induction and maintenance therapy for Crohn’s disease. N. Engl. J. Med..

[25] Sands B.E., Feagan B.G., Rutgeerts P., Colombel J.F., Sandborn W.J., Sy R., D’Haens G., Ben-Horin S., Xu J., Rosario M. (2014). Effects of vedolizumab induction therapy for patients with Crohn’s disease in whom tumor necrosis factor antagonist treatment failed. Gastroenterology.

[26] Singh S., Dulai P.S., Vande Casteele N., Battat R., Fumery M., Boland B.S., Sandborn W.J. (2019). Systematic review with meta-analysis: Association between vedolizumab trough concentration and clinical outcomes in patients with inflammatory bowel diseases. Aliment. Pharmacol. Ther..

[27] Steenholdt C., Lorentsen R.D., Petersen P.N., Widigson E.S., Kloft C., Klaasen R.A., Brynskov J. (2024). Therapeutic drug monitoring of vedolizumab therapy in inflammatory bowel disease. J. Gastroenterol. Hepatol..

[28] Alsoud D., Vermeire S., Verstockt B. (2020). Monitoring vedolizumab and ustekinumab drug levels in patients with inflammatory bowel disease: Hype or hope?. Curr. Opin. Pharmacol..

