# Supplementary material for: Review and External Evaluation of Population Pharmacokinetic Models for Vedolizumab in Patients with Inflammatory Bowel Disease: Assessing Predictive Performance and Clinical Applicability
Source: Biomedicines. 2024 Dec 27;13(1):43. doi: 10.3390/biomedicines13010043 (PMC11762475; doi:10.3390/biomedicines13010043)
Supplement: Supplementary file 1 [file biomedicines-13-00043-s001.zip › biomedicines-3371625-supplementary.pdf]

Supplementary Material

Review and External Evaluation of Population Pharmacokinetic Models for Vedolizumab in Patients with Inflammatory Bowel Disease: Assessing Predictive Performance and Clinical Applicability

Marija Jovanović[1], Ana Homšek[1], Srđan Marković[2,3], Đorđe Kralj[2], Petar Svorcan[2,3], Tamara Knežević Ivanovski[2], Olga Odanović[2], Katarina Vučićević[1]*

[1]Department of Pharmacokinetics and Clinical Pharmacy, Faculty of Pharmacy, University of Belgrade, 11221 Belgrade, Serbia

[2]Department of Gastroenterology and Hepatology, University Hospital Medical Center "Zvezdara", 11000 Belgrade, Serbia

[3]Faculty of Medicine, University of Belgrade, 11000 Belgrade, Serbia

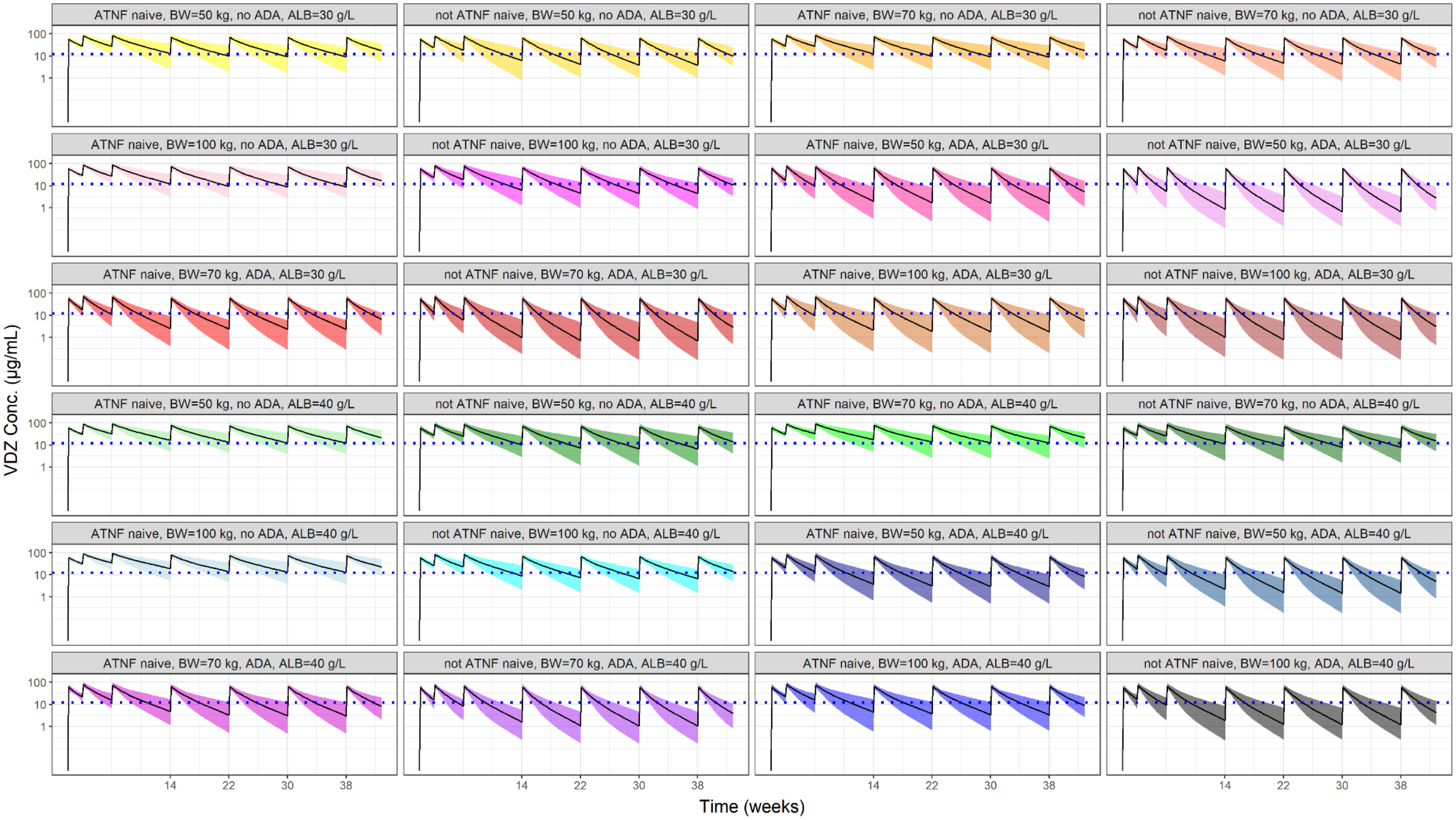

Figure S1. Simulated vedolizumab (VDZ) concentrations with various combination of covariates (ALB=albumin level, ADA= anti-drug antibody status, BW=body weight, ATNF naive=no previous biologic therapy) for dosing regimen (300 mg, dosing frequency Q8W) for model by Hanzel et al. 2022 [14].

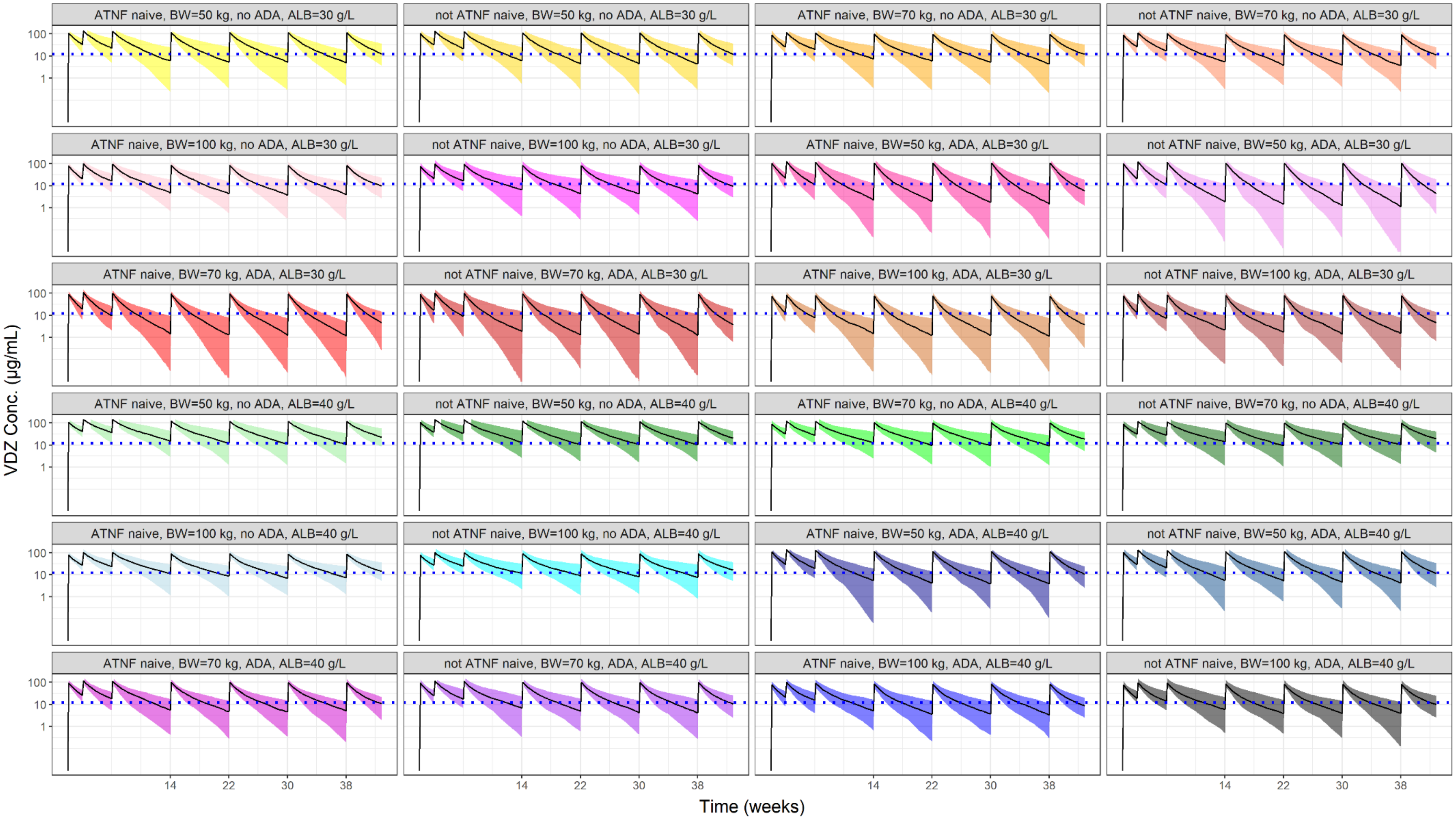

Figure S2. Simulated vedolizumab (VDZ) concentrations with various combination of covariates (ALB=albumin level, ADA= anti-drug antibody status, BW=body weight, ATNF naive=no previous biologic therapy) for dosing regimen (300 mg, dosing frequency Q8W) for model by Okamoto et al. [3]

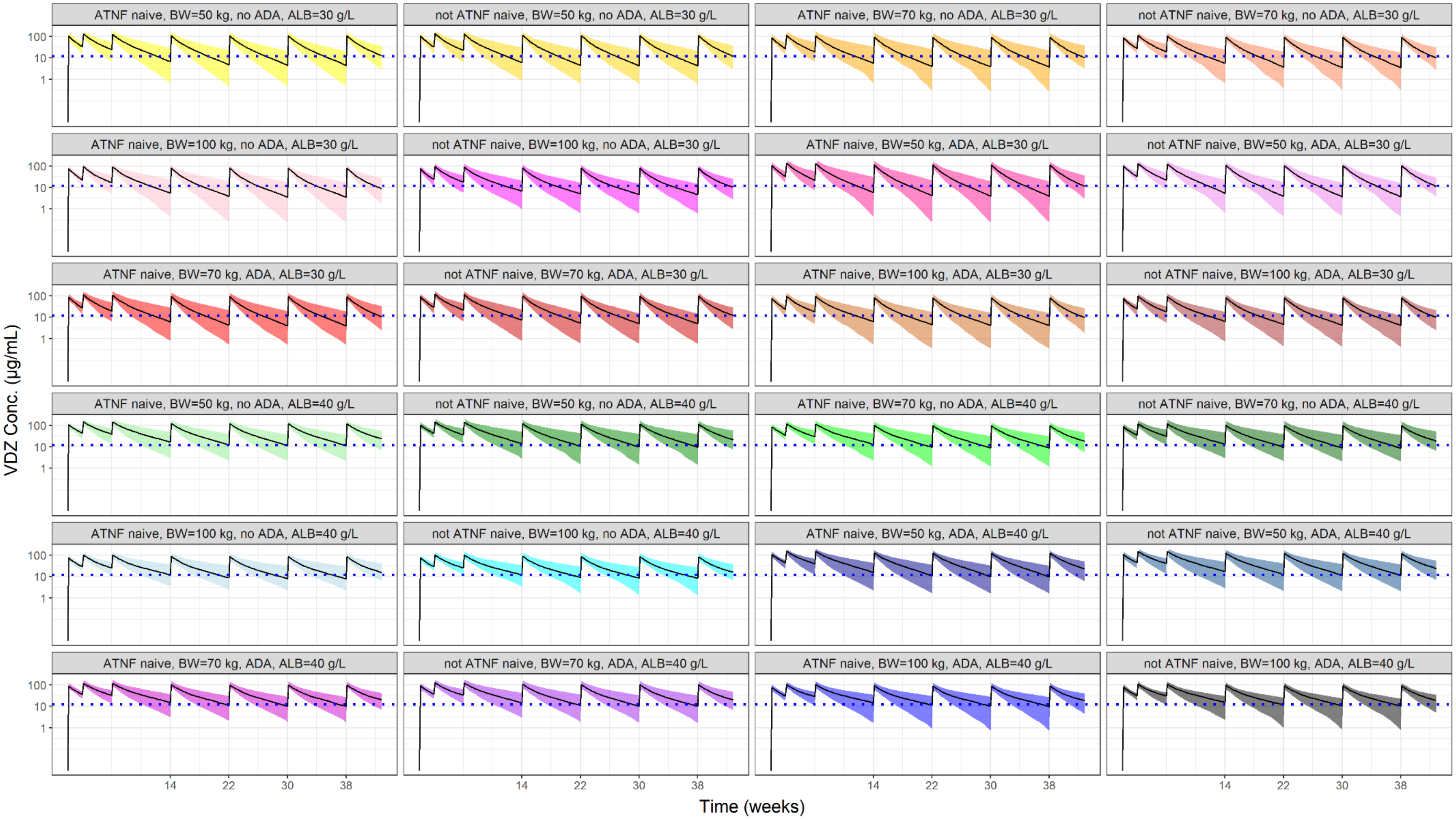

Figure S3. Simulated vedolizumab (VDZ) concentrations with various combination of covariates (ALB=albumin level, ADA= anti-drug antibody status, BW=body weight, ATNF naive=no previous biologic therapy) for dosing regimen (300 mg, dosing frequency Q8W) for model by Rosario et al. [11]
